# From novice to expert: the reproducibility of 3D echocardiographic right ventricular assessment in heart failure patients

**DOI:** 10.3389/fcvm.2025.1523338

**Published:** 2025-08-28

**Authors:** Hayat Memis, Sorina Mihaila-Baldea, Diana Mihalcea, Adriana Andreescu, Diana Dodita, Beatrice Spataru, Andreea Elena Velcea, Alina Nicula, Dragos Vinereanu

**Affiliations:** ^1^ University of Medicine and Pharmacy “Carol Davila”, Bucharest, Romania; ^2^ Department of Cardiology and Cardiovascular Surgery, University and Emergency Hospital of Bucharest, Bucharest, Romania

**Keywords:** heart failure, right ventricle dysfunction, reproducibility, reliability, three-dimensional echocardiography, beginner

## Abstract

**Background:**

Right ventricular (RV) function is a key prognostic factor in patients with heart failure with mildly reduced (HFmrEF) or reduced ejection fraction (HFrEF). While two-dimensional echocardiography (2DE) is used due to its availability, three-dimensional echocardiography (3DE) provides more reproducible measurements, though its use is limited by training requirements.

**Objective:**

To assess whether cardiologists experienced in 2DE with limited 3DE exposure can obtain feasible and reproducible 3DE measurements of RV size and function after a short training in patients with HFmrEF/HFrEF.

**Methods:**

161 patients hospitalized for decompensated HFmrEF/HFrEF (mean age 58 ± 17 years, 71% males, 3D LVEF 35 ± 10%) were analyzed in the study using 2DE and 3DE assessments. Measurements were performed by an Expert in 2DE and 3DE, and by a Beginner with experience in 2DE but only three months of practical training in 3DE. Measurements were taken at baseline (T0) and after three months of practical training in 3DE (T1) to assess intra- and inter-observer reproducibility.

**Results:**

The study demonstrated high intra-observer reproducibility for 2DE parameters by the Beginner with 95% ICCs of: 0.98 (0.98–0.99) for RV diameter, 0.97 (0.94–0.98) for TAPSE, 0.92 (0.90–0.99) for RVFAC, 0.96 (0.95–0.98) for S’, and 0.98 (0.97–0.99) for RVFWS. Conversely, there was a slightly lower inter-observer reproducibility compared to the Expert for the same 2D parameters, with ICCs of: 0.81 (0.71–0.87) for RV diameter, 0.91 (0.88–0.94) for TAPSE, 0.86 (0.81–0.90) for RVFAC, 0.90 (0.88–0.93) for S’, and 0.93 (0.85–0.96) for RVFWS, respectively. The Beginner's intra-observer reproducibility for 3DE parameters was good at baseline, after short theoretical training in 3DE, with ICCs of: 0.87 (0.83–0.91) for RVEDV, 0.85 (0.79–0.89) for RVESV, and 0.90 (0.87–0.93) for RVEF, respectively, and improved significantly after 3 months of practice in 3DE, with ICCs of: 0.96 (0.92–0.97) for RVEDV, 0.95 (0.94–0.98) for RVESV, and 0.95 (0.91–0.97) for RVEF. Bland-Altman analysis showed no systematic bias between the Expert and Beginner for both 2DE and 3DE measurements, confirming the robustness of 3DE across different experience levels.

**Conclusions:**

After brief training, 2DE-proficient cardiologists can perform accurate and reproducible 3DE measurements of RV function, supporting broader clinical use of 3DE in heart failure assessment.

## Introduction

Although, for many years, it was considered a simple conduit linking the venous and pulmonary circulation, the right ventricle (RV) plays an essential role in cardiac physiology (1). Nowadays, the assessment of RV size and function is an important predictor of morbidity and mortality in cardiovascular disease (2). In recent years, RV dysfunction has established itself as an independent prognostic factor for mortality and morbidity in a wide spectrum of cardiovascular diseases such as heart failure with reduced or preserved left ventricular ejection fraction (HFrEF or HFpEF), arrhythmogenic right ventricular cardiomyopathy, ischemic heart disease, valvular heart disease, cardiomyopathies, pulmonary hypertension, arrhythmogenic RV cardiomyopathy, heart transplantation, and even among COVID-19 patients (3–11). Therefore, an accurate characterization of RV remodeling and dysfunction using feasible and reproducible imaging methods is essential to ensure timely and efficient management of these patients.

Describing RV anatomy and quantifying RV function using non-invasive techniques is considered a challenging task due to its complex shape and anatomy. Currently, the most commonly used method is two-dimensional echocardiography (2DE) due to its availability and cost-effectiveness. The parameters most commonly measured by 2DE include tricuspid annulus plane systolic excursion (TAPSE), the peak systolic velocity of tissue Doppler imaging-derived tricuspid annular lateral systolic velocity (S'), RV end-diastolic and end-systolic areas (RVEDA and RVESA), and derived RV fractional change (RVFAC). However, these parameters are prone to errors due to the RV's complex anatomy, including its inflow and outflow compartments that give it a unique “bagpipe” shape, and its anterior position in the mediastinum, which increases cut-plane variability (12, 13). RV hyper-trabeculation makes endocardial tracing challenging, resulting in less reproducibility (13). In contrast, 3DE may overcome these geometric challenges by creating full-volume datasets, allowing measurement of RV end-diastolic (RVEDV) and end-systolic volumes (RVESV), and RV ejection fraction (RVEF). Although cardiac magnetic resonance (CMR) remains the gold standard for RV evaluation, it is time-consuming, expensive, and partially limited by the presence of cardiac devices. In particular, right ventricular function is a critical determinant of outcomes in patients undergoing left ventricular assist device (LVAD) implantation, where RV failure remains a major complication and prognostic factor (14–16). Therefore, a more cost-effective method with good feasibility, reproducibility, and a short learning curve would be beneficial to improve patient evaluation.

### **Aims** of the study

This study aimed to assess whether cardiologists with advanced training in 2DE, but only introductory experience in 3DE, can obtain feasible and reproducible measurements of right ventricular size and function using 3DE after a brief period of focused training, in patients with HFmrEF and HFrEF.

## Materials and methods

### Study population

One hundred and eighty-one consecutive patients hospitalized for decompensated heart failure with mildly reduced or reduce ejection fraction (HFmrEF/HFrEF) according to current ESC guidelines (13), with a broad spectrum of left and right ventricular dysfunction severities, were referred to a high-flow echocardiography laboratory between September 2020 and December 2022. Inclusion criteria were: hospitalization for decompensated HFmrEF or HFrEF (NYHA class II-IV); LVEF < 50% by 2DE-using biplane Simpson's method; regular cardiac rhythm; optimal focus echocardiographic images. Exclusion criteria included: age under 18 years; inability to sign informed consent or comprehend the protocol; acute coronary syndrome; myocarditis; arrhythmia; severe organic valvular heart diseases; congenital heart disease; pulmonary hypertension; poor 2D or 3D echocardiography images. The study finally included 161 patients that underwent scheduled comprehensive transthoracic 2DE and 3DE acquisitions based on a predefined research protocol, when they were hemodynamically stable and able to cooperate to the examination. The study protocol was approved by the Ethics Committee of the University and Emergency Hospital of Bucharest (approval no. 137/2020).

### Clinical findings

Clinical findings were recorded at the time of echocardiographic acquisition, including age, gender, weight, height, body surface area (BSA), blood pressure (BP), and admission diagnosis to the Cardiology Department.

### Echocardiography

A cardiologist with advanced experience in 2D and 3DE (over 10 years) was named “Expert” (SMB), and she conducted echocardiography acquisitions of 2D and 3DE datasets in all patients. A commercially available echo machine (Vivid E95, GE Vingmed, Horten, NO) equipped with standard probes for 2D (M5S) and 3D (4 V) was used. The acquisition protocol followed current guideline recommendations (13). Afterwards, the Expert measured the 2D and 3DE datasets in all patients.

Another cardiologist (HM) with advanced experience in 2DE (more than 5 years) and a beginner level of training in 3DE (one month of theoretical training in 3DE) was named “Beginner”. The Beginner performed the measurements of 2DE and 3DE parameters for RV size and function in a blinded fashion, two times: at baseline (T0), immediately after the one-month theoretical training, and at T1 (after 3 months of practicing 3DE in the echocardiography laboratory).

**Figure 1:**
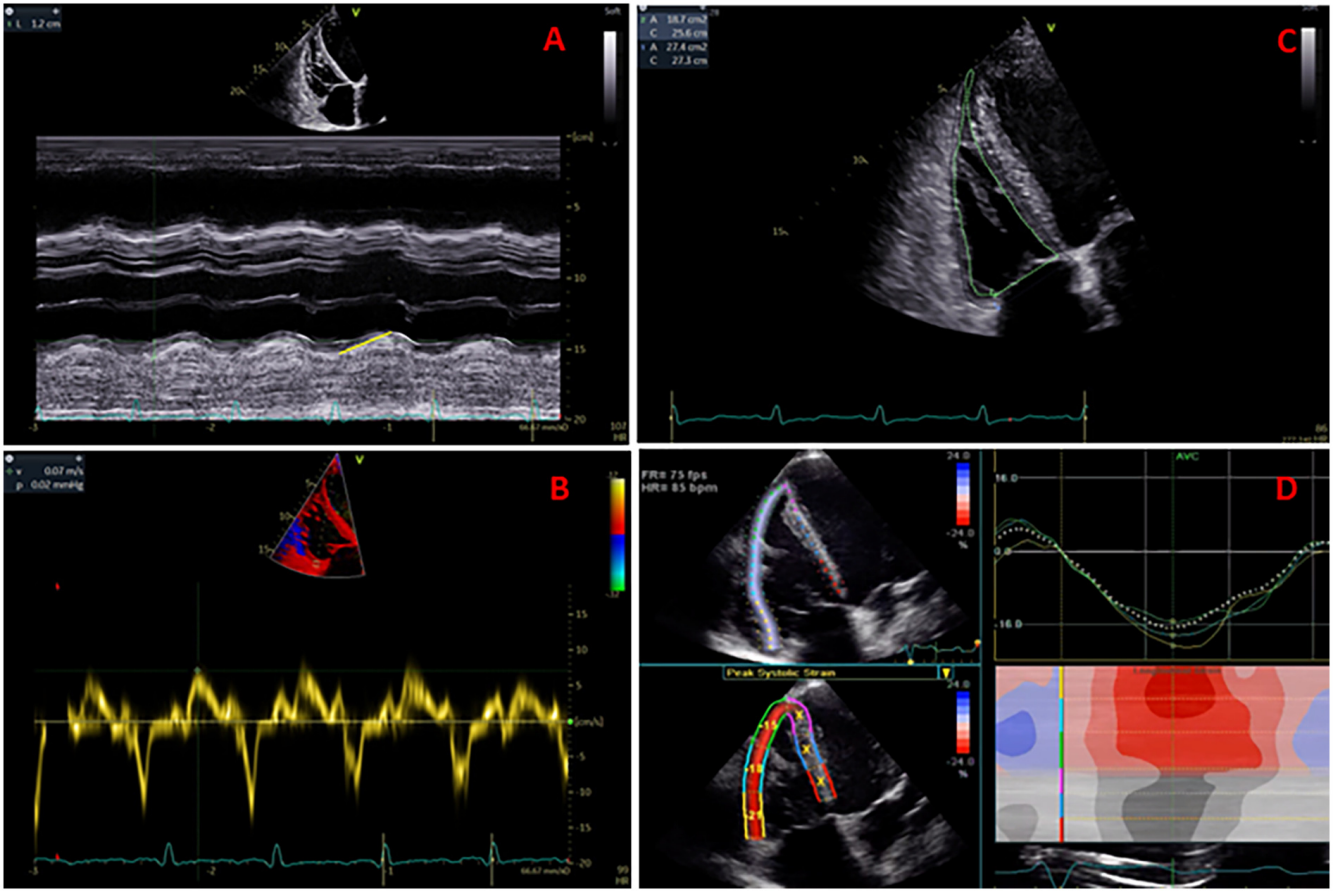
Measurement of TAPSE (panel **A**), S' velocity by TDI (panel **B**), RV areas and fractional area change (panel **C**), and RV free wall longitudinal strain (panel **D**).

2DE and 3DE images were obtained from dedicated four-chamber RV views. The measured 2DE parameters of RV size and function were: RV end-diastolic diameter (RV diameter), RV end-diastolic and end-systolic areas (RVEDA and RVESA), RV fractional area change (RVFAC), tricuspid annular plane systolic excursion (TAPSE), RV free wall Tissue Doppler systolic velocity (S'), and RV free wall longitudinal strain (RVFWLS) (Figure 1).

**Figure 2:**
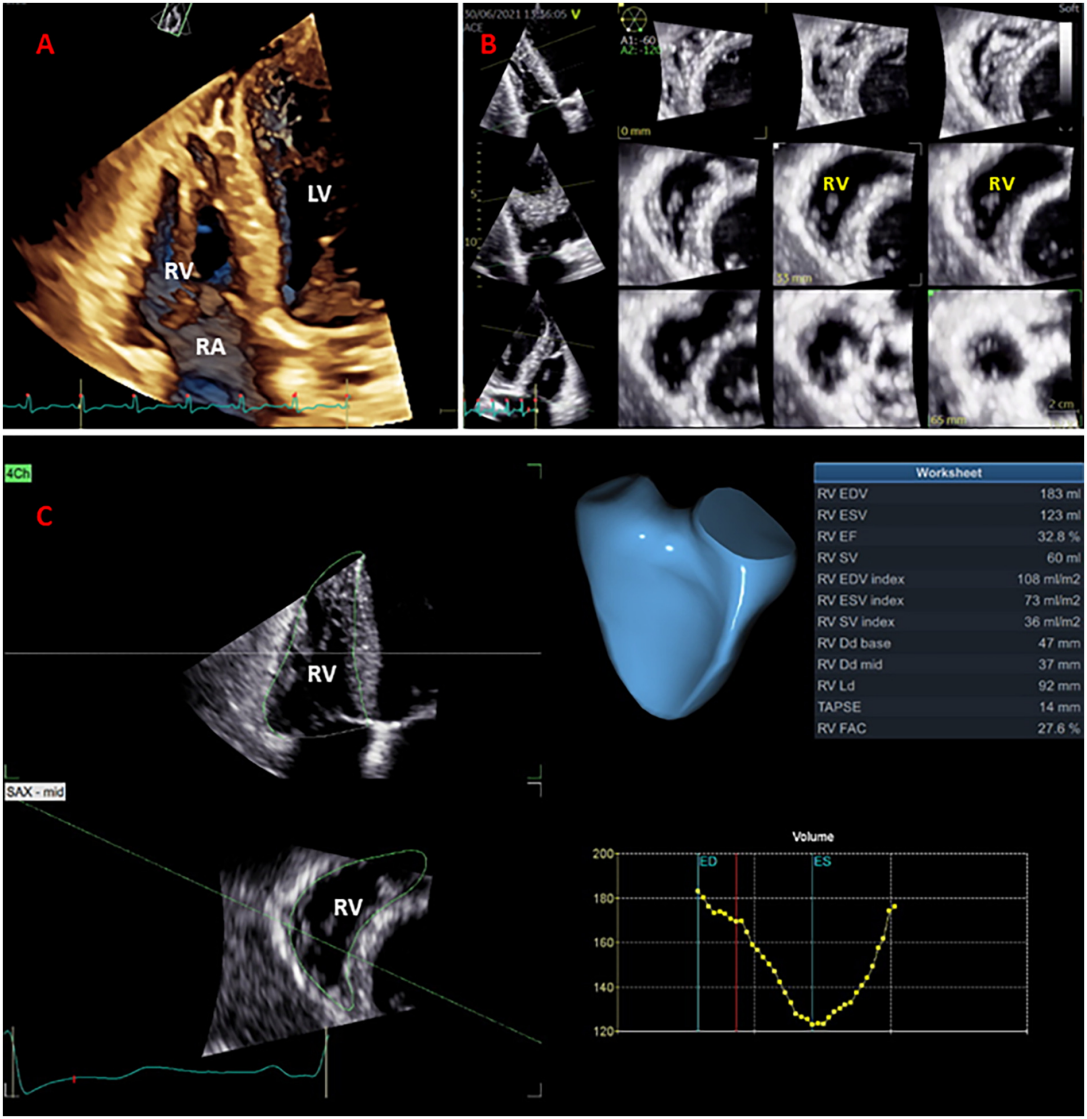
Right ventricular (RV) full-volume (panel **A**) and multi-slice display (panel **B**) used for qualitative and complete analysis of the RV volumes and ejection fraction (panel **C**).

A semi-automated software package for RV (GE 4D auto RVQ) was used to measure the 3D datasets. The workflow for RV size and function assessment initiated with semi-automated detection of RV endocardial borders, followed by manual tracing to optimize endocardial borders during end-diastole and end-systole phases of the cardiac cycle, using electrocardiographic gating. Both the Expert and Beginner excluded patients with suboptimal 3DE views. The measured 3DE parameters of RV size and function were: RV end-diastolic and end-systolic volumes (RVEDV and RVESV), RV ejection fraction (RVEF), 3D RVFAC, and 3D TAPSE (Figure 2). The GE 4D Auto RVQ software used during this study has since been updated; newer versions may offer enhanced automation and precision.

### Statistics

Continuous data are presented as mean ± SD, and categorical data as frequency and percentages (%). Measurements obtained by the Expert at T0 and by the Beginner at T0 and T1 were compared using Student *T*-test analysis. Intra-observer and inter-observed reproducibility of measurements were assessed using intra-class coefficients (ICCs), with an ICC < 0.5 considered poor, 0.5 ≤ ICC < 0.75 considered moderate, 0.75 ≤ ICC < 0.90 considered good, and an ICC ≥ 0.90 considered excellent. Agreements between measurements performed by the two investigators were analyzed using Bland Altman plots and presented as mean bias ± 95% limits of agreement (LOA). A *p*-value less than 0.5 was considered significant. Data analysis was performed using statistical software packages (SPSS 20, SPSS Inc., Chicago, Illinois, and Prism GraphPad Software version 10).

## Results

One hundred and eighty patients hospitalized for decompensated HFmrEF or HFrEF were initially enrolled in the study. From the initial cohort, 5 patients were excluded due to inadequate 2DE, and 14 patients were excluded due to poor 3DE images or inability to follow breath-hold indications necessary for multi-beat 3DE acquisitions (resulting in 97% and 92% feasibility of 2DE and 3DE acquisition and analysis, respectively). Table 1 provides the general characteristics of the remaining study population of 161 patients (58 ± 17 years, 71% male, 3D LVEF of 35 ± 10%, and 3D RVEF of 37 ± 10% respectively).

**Table 1:** General characteristics of the study population.

Parameter	Mean ± SD
Number of patients	161
Age (yrs)	58 ± 17
Gender	
Males (%)	71
Females (%)	29
BSA (m^2)^	1.96 ± 0.2
HR (bpm)	78 ± 15
SBP (mmHg)	125 ± 19
DBP (mmHg)	57 ± 15
3D LVEF (%)	35 ± 10
2D RV diameter (mm)	35 ± 7
2D RVEDA (cm^2^)	20 ± 6
2D RVESA (cm^2^)	13 ± 6
2D RVFAC (%)	36 ± 12
3D RVEDV (ml)	184 ± 63
3D RVESV (ml)	118 ± 52
3D RVEF (%)	37 ± 10

BSA, body surface area; DBP, diastolic blood pressure; HR, heart rate; LVEF, left ventricular ejection fraction; RVEDV, right ventricular end-diastolic volume; RVEF, right ventricular ejection fraction; RVEDA, right ventricular end-diastolic area; RVESA, right ventricular end-systolic area; RVESV, right ventricular end-systolic volume; RVFAC, right ventricular fractional area change; SBP, systolic blood pressure.

The average resolution was 59 ± 9 frames per second for 2DE datasets and 25 ± 6 volumes per second for 3DE datasets. The 2DE-measured parameters were compared between the Expert and Beginner at T0 initially, to assess the inter-observer reproducibility, and between the Beginner at T0 and T1 subsequently to assess intra-observer reproducibility. The 3DE-measured parameters were compared between the Expert and Beginner at T0 initially, and the Beginner at T1 subsequently, to assess the evolution of the inter-observer reproducibility for the 3DE assessment of the RV function.

### Duration of the echocardiographic assessment

When compared, the mean analysis times of the Expert and the Beginner were: 64 ± 8 vs. 66 ± 5 s for the measurement of RV diameter, RV areas, and RVFAC; 70 ± 10 vs. 73 ± 12 s for the measurement of RV S' and RVFWLS; and 110 ± 13 vs. 114 ± 22 s for the measurement of the 3D RV volumes, RVEF, and RVFAC (all *p* > 0.05).

### Right ventricular assessment by two-dimensional echocardiography

The Beginner user demonstrated good intra-observer reproducibility for the 2DE-measured parameters performed at T0 and T1, with 95% ICCs of: 0.98 (0.97–0.99) for TA diameter, 0.98 (0.98–0.99) for 2D RV diameter, 0.97 (0.94–0.98) for 2D TAPSE, 0.92 (0.90–0.99) for 2D RVFAC, 0.96 (0.95–0.98) for S', and 0.98 (0.97–0.99) for RVFWS, respectively (Table 2). In contrast, lower inter-observer reproducibility was noted for the 2DE-measured parameters between the Expert and Beginner T0 users, with 95% ICCs of: 0.91 (0.88–0.94) for TA diameter, 0.81 (0.71–0.87) for 2D RV diameter, 0.91 (0.88–0.94) for 2D TAPSE, 0.86 (0.81–0.90) for 2D RVFAC, 0.90 (0.88–0.93) for S', and 0.93 (0.85–0.96) for RVFWS, respectively (Table 2).

**Table 2:** Intra-observer and inter-observer reproducibility between beginner and expert users in 2D measurements of the right ventricle volumes and ejection fraction.

2DE parameters	Expert (Mean ± SD)	Beginner T0 (Mean ± SD)	Beginner T1 (Mean ± SD)	Beginner T0 vs. Beginner T1 ICC (95% CI) Intra-observer reproducibility	Expert vs. Beginner T0 ICC (95% CI) Inter-observer reproducibility
TA diameter (mm)	31 ± 6.5	32 ± 6.1	31 ± 1.5	0.98 (0.97–0.99)	0.91 (0.88–0.94)
RV diameter (mm)	35 ± 7.0	36 ± 7.2	37 ± 1.4	0.98 (0.98–0.99)	0.81 (0.71–0.87)
TAPSE (mm)	18 ± 3.8	19 ± 3.2	18 ± 1.2	0.97 (0.94–0.98)	0.91 (0.88–0.94)
RVEDA (cm^2^)	20 ± 6.0	22 ± 7.2	22 ± 2.8	0.95 (0.92–0.97)	0.80 (0.70–0.86)
RVESA (cm^2^)	13 ± 5.8	15 ± 6.6	14 ± 2.2	0.94 (0.89–0.96)	0.81 (0.70–0.87)
RVFAC (%)	37 ± 14.0	35 ± 11.0	36 ± 2.7	0.92 (0.90–0.99)	0.86 (0.81–0.90)
S’ (cm/s)	11 ± 3.5	10 ± 3.0	11 ± 4.8	0.96 (0.95–0.98)	0.90 (0.88–0.93)
RVFW strain (%)	19 ± 8.1	17 ± 7.8	18 ± 2.2	0.98 (0.97–0.99)	0.93 (0.85–0.96)

RV, right ventricle; RVEDA, right ventricular end-diastolic area; RVESA, right ventricular end-systolic area; RVFAC, right ventricular fraction area change; RVFWS, right ventricular free wall strain; S’, systolic velocity of the right ventricular free wall measured by Tissue Doppler Imaging; TA, tricuspid annulus; TAPSE, tricuspid annulus plane systolic excursion.

### Right ventricular assessment by three-dimensional echocardiography

The Beginner user presented lower inter-observer reproducibility for the 3DE-measured parameters at T0 (after one month theoretical training) when compared with the Expert, with ICCs of: 0.95 (0.92–0.97) for 3D TA diameter, 0.87 (0.82–0.91) for 3D RV mid diameter, 0.87 (0.83–0.91) for RVEDV, 0.85 (0.79–0.89) for RVESV, and 0.90 (0.87–0.93) for 3D RVEF, respectively (Table 3). However, after three months of practice in the echocardiography laboratory, increased inter-observer reproducibility was observed for the 3DE parameters between the Expert and Beginner users at T1, with 95% ICCS of: 0.92 (0.89–0.94) for 3D TA diameter, 0.92 (0.87–0.96) for 3D RV mid diameter, 0.96 (0.92–0.97) for RVEDV, 0.95 (0.94–0.98) for RVESV, and 0.95 (0.91–0.97) for 3D RVEF, respectively (Table 3).

**Table 3:** Reproducibility of the 3D measurements of the right ventricle volumes and ejection fraction provided by the expert user and the beginner user before (T0) and after the training (T1), respectively.

3DE parameters	Expert (Mean ± SD)	Beginner T0 (Mean ± SD)	Beginner T1 (Mean ± SD)	Expert vs. Beginner T0 ICC (95% CI)	Expert vs. Beginner T1 ICC (95% CI)	Beginner T0 vs. Beginner T1 ICC (95% CI)
TA diameter (mm)	49 ± 9.8	51 ± 8.8	50 ± 3.4	0.95 (0.92–0.97)	0.92 (0.89–0.94)	0.92 (0.90–0.93)
RV mid diameter (mm)	37 ± 9.0	38 ± 8.6	38 ± 3.8	0.87 (0.82–0.91)	0.92 (0.87–0.96)	0.88 (0.84–0.90)
RVEDV (ml)	184 ± 62.7	181 ± 6.3	188 ± 6.4	0.87 (0.83–0.91)	0.96 (0.92–0.97)	0.89 (0.85–0.92)
RVESV (ml)	118 ± 52.0	113 ± 5.1	119 ± 4.9	0.85 (0.79–0.89)	0.95 (0.94–0.98)	0.87 (0.82–0.92)
RVEF (%)	37 ± 9.6	38 ± 9.1	37 ± 3.6	0.90 (0.87–0.93)	0.95 (0.91–0.97)	0.91 (0.88–0.94)
TAPSE (mm)	15 ± 4.6	16 ± 4.6	15 ± 2.7	0.74 (0.73–0.80)	0.88 (0.83–0.92)	0.78 (0.75–0.84)

TA, tricuspid annulus; RV, right ventricle; RVEDA, right ventricular end-diastolic area; RVESA, right ventricular end-systolic area; RVEF (%), right ventricular ejection fraction; TAPSE, tricuspid annulus plane systolic excursion.

### Bias and limits of agreement

**Figure 3:**
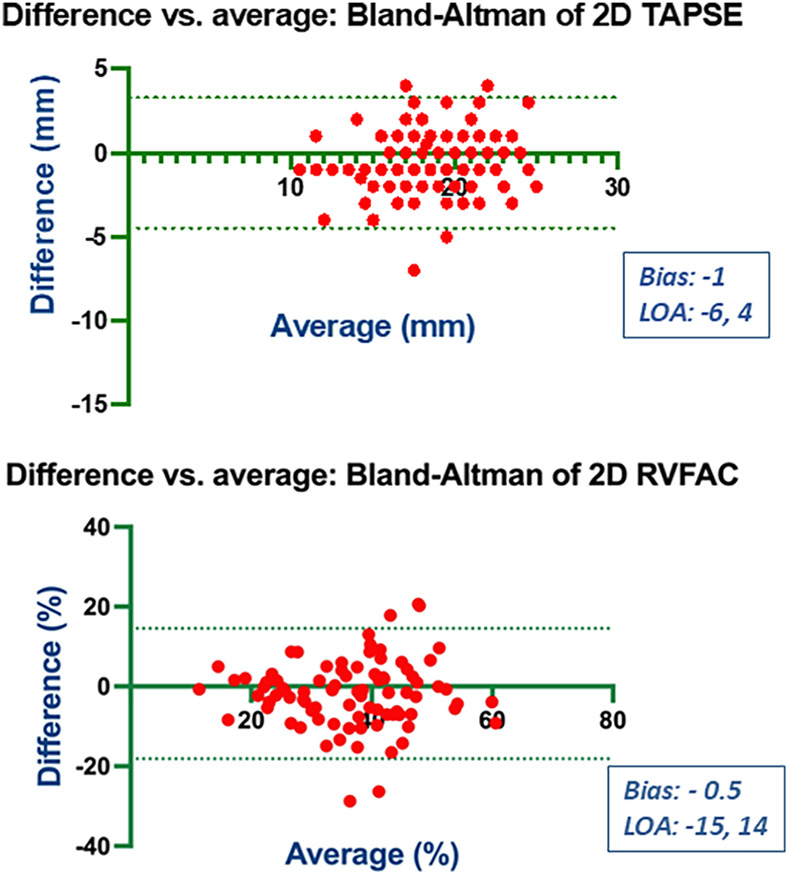
Bland Altman plots for the measurement of TAPSE and RVFAC compared between the expert and the beginner at T1.

**Figure 4:**
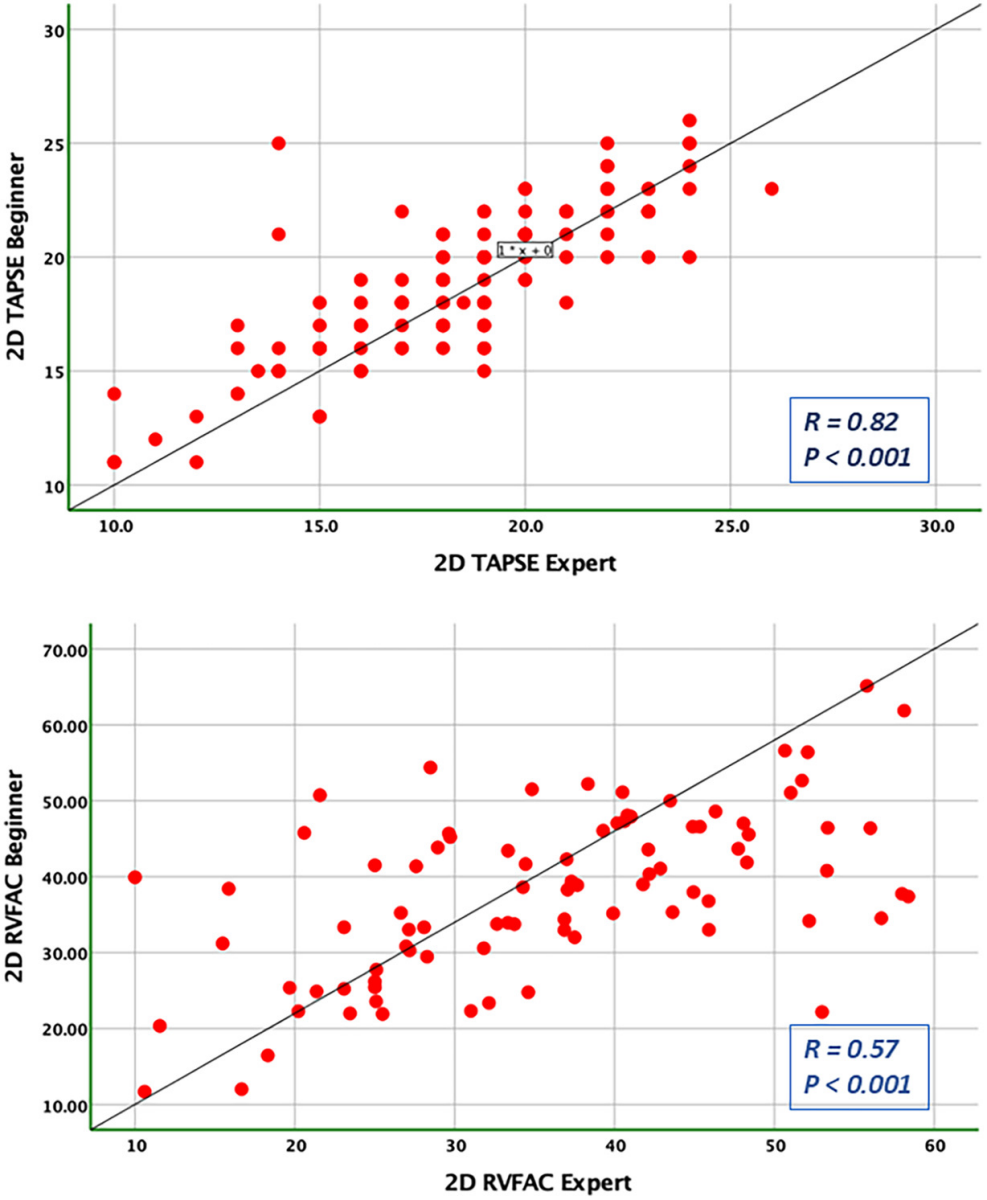
Pearson correlation plots for the measurement of TAPSE and RVFAC compared between the expert and the beginner at T1.

**Figure 5:**
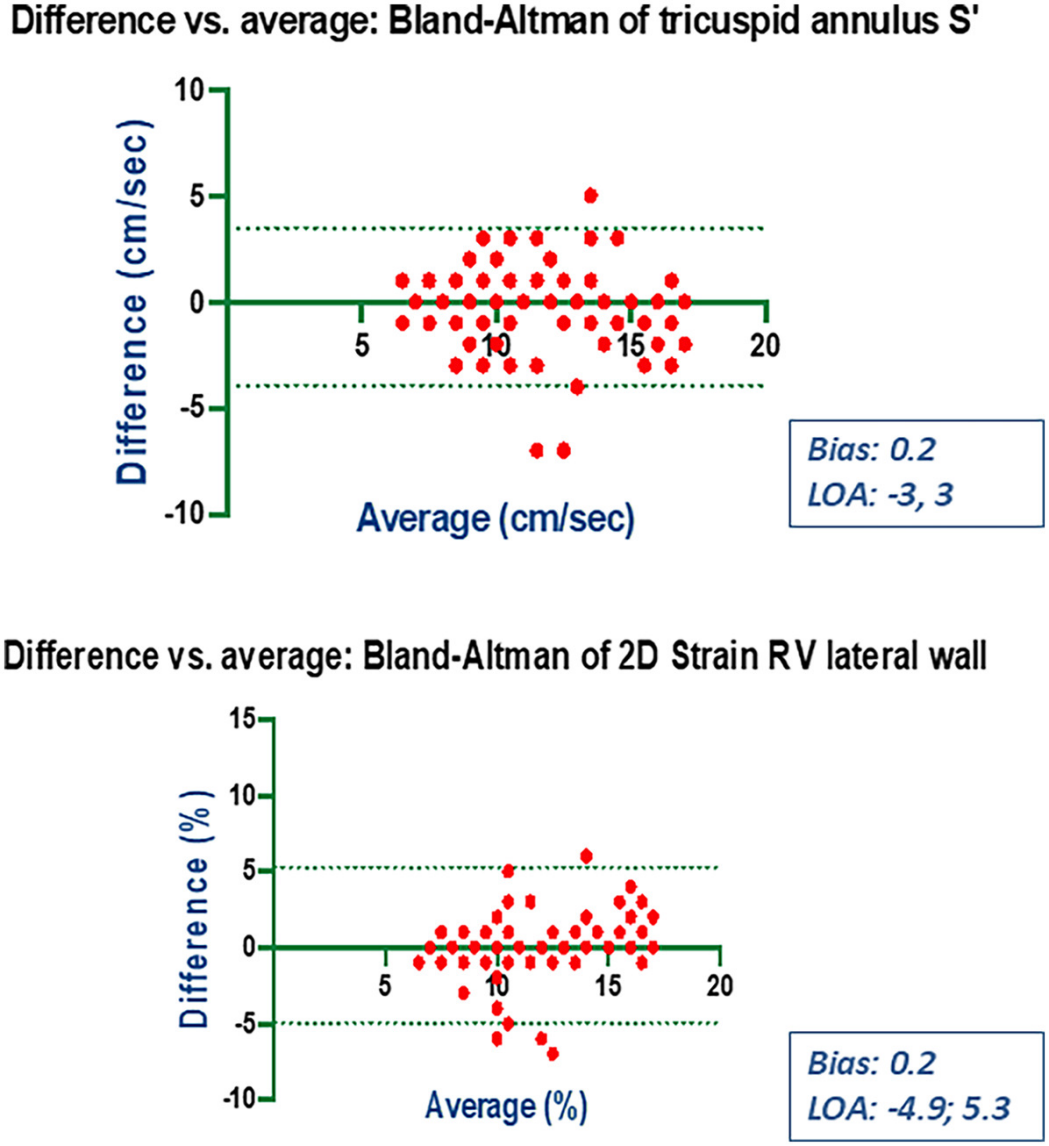
Bland Altman plots for the measurement of S’ velocity by TDI and RV longitudinal strain compared between the expert and the beginner at T1.

**Figure 6:**
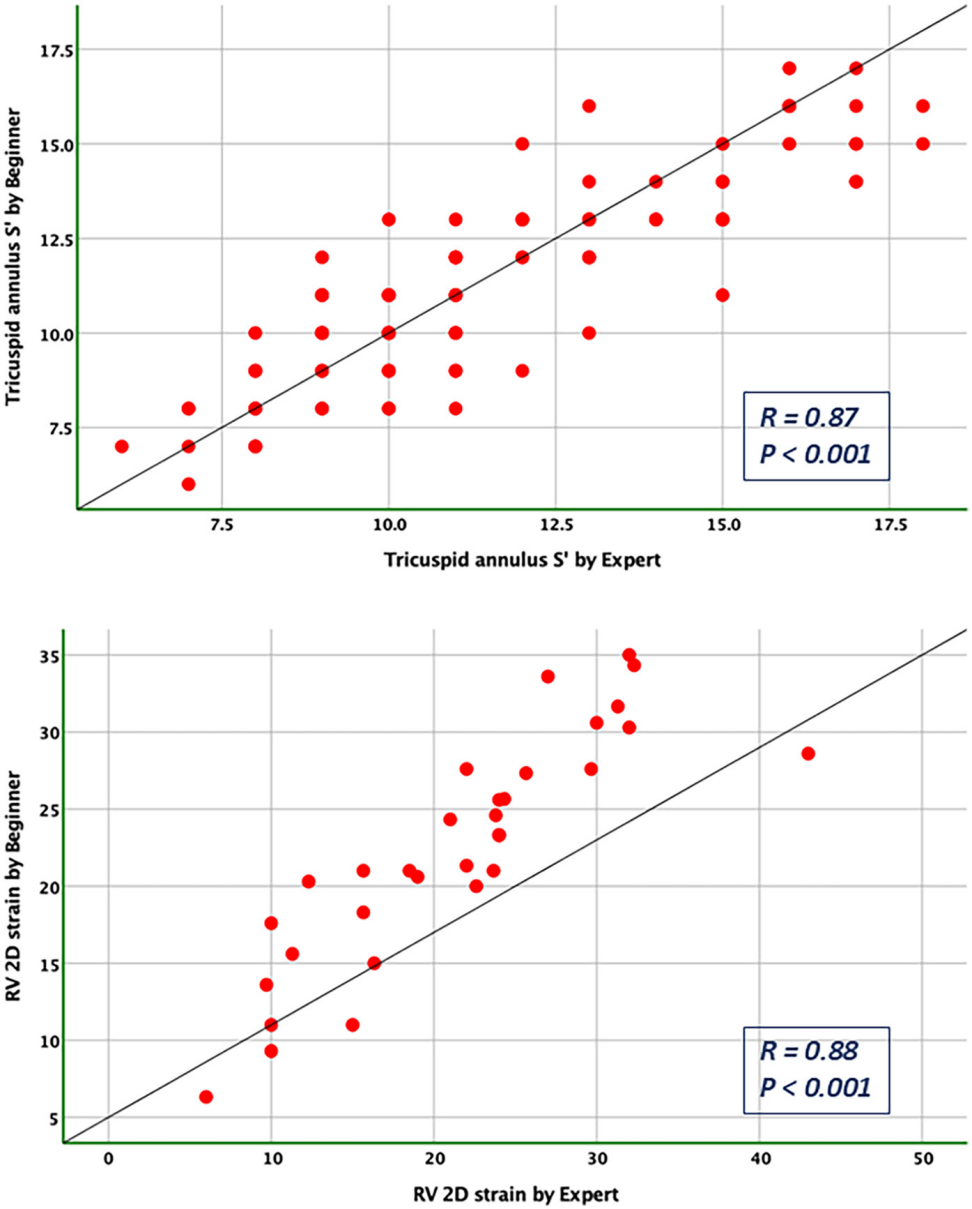
Pearson correlation plots for the measurement of S’ velocity by TDI and RV longitudinal strain compared between the expert and the beginner at T1.

**Figure 7:**
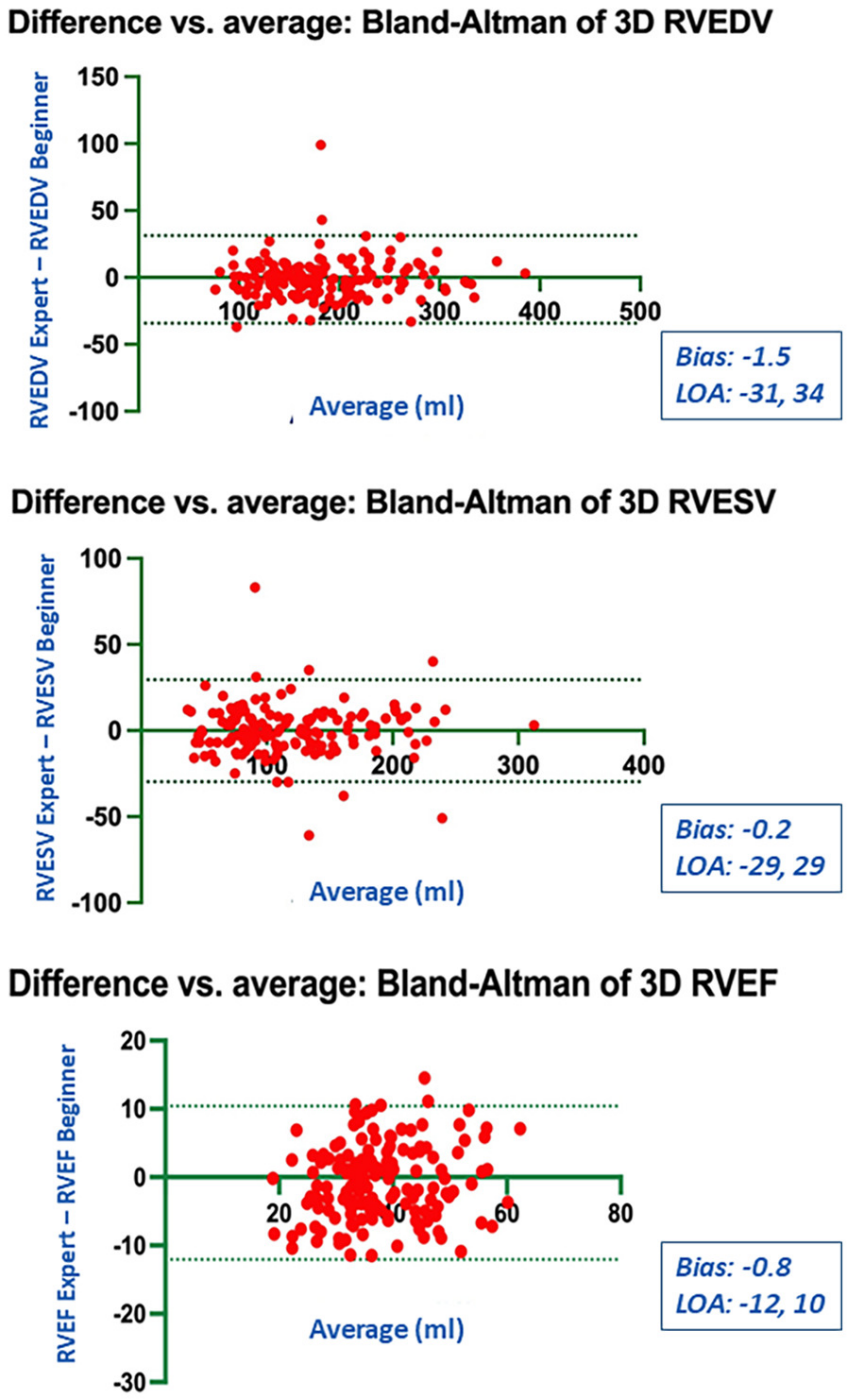
Bland Altman plots for the measurements of RV end-diastolic and end-systolic volumes and RV ejection fraction compared between the expert and the beginner at T1.

**Figure 8:**
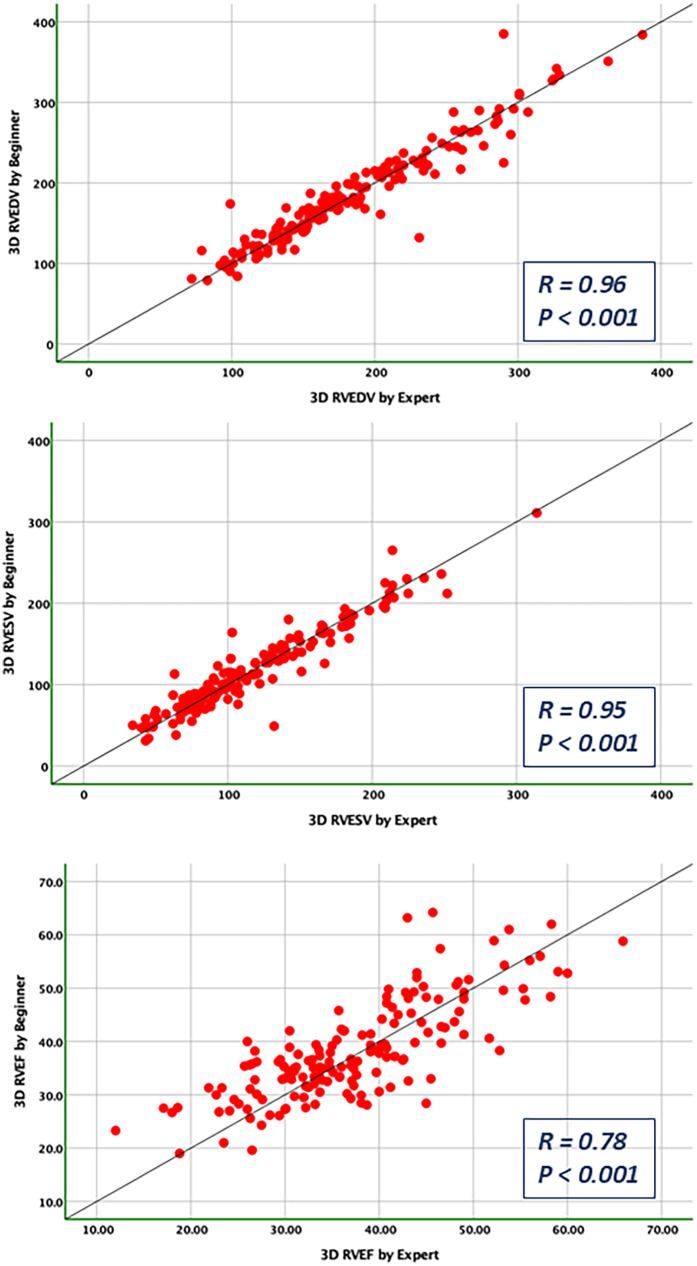
Pearson correlations for the measurements of RV end-diastolic and end-systolic volumes and RV ejection fraction compared between the expert and the beginner at T1.

Bland-Altman analysis revealed no systematic bias between measurements performed by Expert and Beginner users for both 2DE and 3DE. Specifically, in 2DE, measurements by both the Expert and the Beginner showed lower limits of agreement (LOA) for TAPSE, RV S', and RVFWLS (Figures 3, 4), while exhibiting higher LOA for RVFAC (Figures 5, 6). Conversely, 3DE measurements by both the Expert and the Beginner user at T1 displayed good LOA for RV volumes and EF, which is particularly noteworthy considering the wide range of RV sizes and functions analyzed in the study (Figures 7, 8).

## Discussions

In our study, the main findings are: (1) cardiologists with 3 months dedicated training in 3DE produced reproducible measurements for RV size and function by comparison with an Expert, suggesting that 3DE can be reliably utilized even by those new to the technique; (2) while initial inter-observer variability was observed, it decreased with increased operator practical experience, highlighting a relatively fast learning curve for 3DE, which is encouraging for its adoption by clinicians with varying levels of expertise in echocardiography; (3) there was no systematic bias between the 3DE-measurements of RV function made by the Expert and the Beginner after training, demonstrating that 3DE can be a robust method for RV evaluation across different levels of operator experience and high range of RV's size and function. These findings support the potential for broader implementation of 3DE in the echocardiographic assessment of the RV size and function, enhancing its utility as a feasible and reliable diagnostic tool in both expert hands and those with less experience.

While 2DE remains prevalent in clinical settings due to its wide availability, its reproducibility has been a subject of previous validation (17–19). The biases identified by us, as well, between the 2DE-measurements provided by the Expert and the Beginner users arise from variations in RV cut-plane selections, despite efforts to achieve optimal visualization. The reproducibility of our 2DE measurements aligns with those previously reported (17, 20). We observed no systematic bias in the agreement between 2DE measurements. However, the differences that did occur were likely caused by the variability in measuring RV dimensions with respect to its hyper-trabeculated structure and difficult delineation of the endocardial borders. TAPSE remains the most accessible and straightforward method for bedside assessment and carries a significant prognostic value in conditions such as coronary artery disease, heart failure, and pulmonary embolism. Our findings indicated good intra-observer reproducibility for 2DE TAPSE, with no systematic bias, but with notable limits of agreement between the Expert and the Beginner. This discrepancy could be due to the variability in choosing TAPSE's slope between different cardiac cycles, which is angle- and heart-movements dependent, underscoring the necessity for additional measures to enhance RV quantification (21).

Conversely, the RV fractional area change (RVFAC) displayed good intra-observer reproducibility but diminished inter-observer agreement, similar with findings from existing literature and underscoring the need for it to supplement other parameters (20, 22, 23). Accurately delineating the hyper-trabeculated endocardial borders of the RV at end-diastole and end-systole presents a significant challenge due to the inherent anatomical complexity and the potential for variability among observers.

Right ventricular free wall systolic velocity (S'), assessed through tissue Doppler imaging, exhibited excellent intra- and inter-observer consistency, outperforming both RVFAC and TAPSE when compared with cardiac magnetic resonance (CMR)- derived RVEF (16, 24). Our study demonstrated that measurements of S' and 2D TAPSE are closely aligned, with S' displaying equivalent reproducibility and outperforming RVFAC in terms of consistency across measurements. Nonetheless, it shares the limitations of TAPSE and RVFAC regarding angle dependency and highlighting only the longitudinal function of the RV.

Advancements in speckle tracking imaging tried to overcome the angle dependency issues, with RV longitudinal strain offering a more comprehensive representation of RV myocardial deformation. This has proven especially useful in detecting early RV dysfunction across various cardiovascular conditions (17, 20, 25). Despite its potential, challenges such as reliance on post-processing and vendor-specific limitations persist (25–27). In our study, there was a good intra- and inter-observer reproducibility for RVFWLS, but with notable LOA, which may be crucial for establishing patients’ risk.

In contrast to 2DE, 3DE provides a more precise characterization of the RV's complex geometry. Although prone to underestimation of RV volumes, 3DE's alignment with CMR assessments has shown progressive refinement and increasing reproducibility, even when comparing novice to experienced operators (28–30). The ability to measure RVEF accurately in labs proficient in 3DE is invaluable, particularly for those patients with a clear acoustic window, potentially positioning 3DE as a precursor to CMR evaluation, which remains the gold standard despite its limitations in availability and patient stability requirements (31, 32). Three-dimensional echocardiography (3DE) is increasingly recognized in guidelines for cardiac chamber quantification due to its superior accuracy and reproducibility, particularly for right ventricular assessment (33). In this context, our study further explores whether such advantages can be retained when 3DE is performed by cardiologists with limited prior experience, following a brief, focused training period (12). Notably, our findings suggest that even cardiologists with basic training in 3DE can achieve reliable measurements, which is significant for clinical practice. While inter-observer variability was initially present for the 3DE-measure parameters, it improved with experience, indicating that proficiency in 3DE can be obtained relatively quickly. Moreover, the Bland-Altman analysis demonstrated no systematic bias between Expert and Beginner measurements in 3DE, sustaining as argument for its broader implementation. These results advocate for the integration of 3DE into routine RV assessments, even in the setting of a wide range of RV sizes and function. underscoring its practicality and potential to augment clinical diagnostics and follow-up, regardless of the operator's experience level. A reduction of the SD for the measurements performed by the Beginner was observed (Tables 2, 3). This finding is primarily attributable to the progressive improvement in measurement consistency following structured training and hands-on practice in 3DE. The extended period of practical exposure led to a marked improvement in intra-observer reliability, as reflected in the higher intraclass correlation coefficients (ICCs) at T1. The more consistent measurements across repeated assessments resulted in reduced SD values. This trend aligns with existing literature demonstrating that structured echocardiographic training enhances both intra- and inter-observer reproducibility.

While overall inter-observer reproducibility improved, TA diameter may require additional training beyond three months to achieve consistency comparable to RV volumes and function. The Beginner's experience improved significantly regarding RV volumetric assessment, while measuring the TA diameter accurately in 3DE may follow a different learning trajectory. However, the decrease in ICCs for TA diameter at T1 reflects the intrinsic dynamic nature of the tricuspid annulus, measurement technique variability, and the distinct learning curve associated with annular dimensions in 3DE. Additionally, RV volumes and RVEF are derived from semi-automated contouring, whereas TA diameter requires manual caliper placement, which might still be influenced by the operator's experience level. 3D TAPSE, another parameter derived from 3DE, indicated notable variability, reflecting the necessity for further validation to establish its correlation with CMR data.

### Study limitations

Our single-center study's scope was narrowed by the homogeneous use of one echocardiography software vendor and the absence of a test-retest analysis. Although image quality markedly influences 3DE outcomes, we mitigated this by selecting patients with optimal 3DE windows and excluding those with atrial fibrillation, a common rhythm disturbance that can affect measurements. Repeated analysis of the same cases could introduce a degree of bias or variability. Therefore, in order to mitigate this potential source of bias, several steps were adopted to ensure the integrity and validity of the results. Firstly, all measurements at T0 and T1 were performed in a blinded fashion by the Beginner, minimizing any influence of prior knowledge on the measurements at T1. Moreover, there was a minimum interval of three months between the initial and follow-up assessments, which provided the Beginner with adequate time for practical training and proficiency in 3DE, reducing the likelihood of performance bias during repeated measurements*.* Additionally, the semi-automated software used (GE 4D Auto RVQ) is no longer the most current tool available for RV quantification, which may affect generalizability of the results.

## Conclusions

3DE has emerged as a practical, rapidly learned, and reliable modality compared to 2DE for evaluating RV size and function across diverse presentations of RV remodeling and dysfunction. Our findings suggest that 3DE may serve as a robust tool for assessment and ongoing monitoring of heart failure patients, even after a short period of theoretical and practical training.

## Data Availability

The original contributions presented in the study are included in the article/Supplementary Material, further inquiries can be directed to the corresponding author.

## References

[1] Semino T Rosa GM Monacelli F Pellicano R Testino G Porto I. Right ventricle: current knowledge of echocardiographic evaluation of this “forgotten” chamber. Minerva Med. (2024) 115(1):45–60. 10.23736/S0026-4806.23.08575-0 36988493

[2] Wang TKM Jellis C. The role of multimodality imaging in right ventricular failure. Cardiol Clin. (2020) 38(2):203–17. 10.1016/j.ccl.2020.01.006 32284097

[3] Schmeißer A Rauwolf T Groscheck T Fischbach K Kropf S Luani B Predictors and prognosis of right ventricular function in pulmonary hypertension due to heart failure with reduced ejection fraction. ESC Heart Fail. (2021) 8(4):2968–81. 10.1002/ehf2.13386 33934536 PMC8318446

[4] Meng Y Zhu S Xie Y Zhang Y Qian M Gao L Prognostic value of right ventricular 3D speckle-tracking strain and ejection fraction in patients with HFpEF. Front Cardiovasc Med. (2021) 8:694365. 10.3389/fcvm.2021.694365 34277743 PMC8278016

[5] Obokata M Reddy YNV Melenovsky V Pislaru S Borlaug BA. Deterioration in right ventricular structure and function over time in patients with heart failure and preserved ejection fraction. Eur Heart J. (2019) 40(8):689–97. 10.1093/eurheartj/ehy809 30544228 PMC7963126

[6] Tadic M Nita N Schneider L Kersten J Buckert D Gonska B The predictive value of right ventricular longitudinal strain in pulmonary hypertension, heart failure, and valvular disease. Front Cardiovasc Med. (2021) 8:698158. 10.3389/fcvm.2021.698158 34222387 PMC8247437

[7] Malik N Mukherjee M Wu KC Zimmerman SL Zhan J Calkins H Multimodality imaging in arrhythmogenic right ventricular cardiomyopathy. Circ Cardiovasc Imaging. (2022) 15(2):e013725. 10.1161/CIRCIMAGING.121.013725 35147040

[8] Moceri P Duchateau N Baudouy D Schouver ED Leroy S Squara F Three-dimensional right-ventricular regional deformation and survival in pulmonary hypertension. Eur Heart J Cardiovasc Imaging. (2018) 19(4):450–8. 10.1093/ehjci/jex163 28637308

[9] Li M Lv Q Sun W Zhang Y Wu C Zhang Y Prognostic value of right ventricular three-dimensional speckle-tracking strain in adult heart transplantation patients. Int J Cardiovasc Imaging. (2023) 39(7):1275–87. 10.1007/s10554-023-02842-w 37027106

[10] Kim J Volodarskiy A Sultana R Pollie MP Yum B Nambiar L Prognostic utility of right ventricular remodeling over conventional risk stratification in patients with COVID-19. J Am Coll Cardiol. (2020) 76(17):1965–77. 10.1016/j.jacc.2020.08.066 33092732 PMC7572068

[11] Amzulescu MS Slavich M Florian A Goetschalckx K Voigt JU. Does two-dimensional image reconstruction from three-dimensional full volume echocardiography improve the assessment of left ventricular morphology and function? Echocardiography. (2013) 30(1):55–63. 10.1111/j.1540-8175.2012.01800.x 22963450

[12] Sheehan F Redington A. The right ventricle: anatomy, physiology and clinical imaging. Heart. (2008) 94(11):1510–5. 10.1136/hrt.2007.132779 18931164

[13] Lang RM Badano LP Mor-Avi V Afilalo J Armstrong A Ernande L Recommendations for cardiac chamber quantification by echocardiography in adults: an update from the American society of echocardiography and the European association of cardiovascular imaging. Eur Heart J Cardiovasc Imaging. (2015) 16(3):233–70. 10.1093/ehjci/jev014. Erratum in: *Eur Heart J Cardiovasc Imaging*. (2016) **17**(4):412. doi: 10.1093/ehjci/jew041. Erratum in: *Eur Heart J Cardiovasc Imaging*. (2016) **17**(9):969. doi: 10.1093/ehjci/jew124. 25712077

[14] Chai JCK Chew JL Amarasekera A Soliman Aboumarie H Tan TC. Echocardiographic parameters of right ventricular size and function associated with right heart failure after durable left ventricular assist device implantation-A systematic review and meta-analysis. Echocardiography. (2025) 42(3):e70119. 10.1111/echo.70119 40033957 PMC11877009

[15] Gramegna M Vandenbriele C Tavazzi G Basir MB Bleakley C Iannaccone M Percutaneous mechanical circulatory support for acute right heart failure: a practical approach. ESC Heart Fail. (2025) 12(4):2652–68. 10.1002/ehf2.15305 40254772 PMC12287799

[16] McDonagh TA Metra M Adamo M Gardner RS Baumbach A Böhm M 2023 Focused update of the 2021 ESC guidelines for the diagnosis and treatment of acute and chronic heart failure: developed by the task force for the diagnosis and treatment of acute and chronic heart failure of the European Society of Cardiology (ESC) with the special contribution of the heart failure association (HFA) of the ESC. Eur J Heart Fail. (2024) 26(1):5–17. 10.1002/ejhf.3024 38169072

[17] Badano LP Addetia K Pontone G Torlasco C Lang RM Parati G Advanced imaging of right ventricular anatomy and function. Heart. (2020) 106(19):1469–76. 10.1136/heartjnl-2019-315178 32620556

[18] Muraru D Onciul S Peluso D Soriani N Cucchini U Aruta P Sex- and method-specific reference values for right ventricular strain by 2-dimensional speckle-tracking echocardiography. Circ Cardiovasc Imaging. (2016) 9(2):e003866. 10.1161/CIRCIMAGING.115.003866 26860970

[19] Haddad F Hunt SA Rosenthal DN Murphy DJ. Right ventricular function in cardiovascular disease, part I: anatomy, physiology, aging, and functional assessment of the right ventricle. Circulation. (2008) 117(11):1436–48. 10.1161/CIRCULATIONAHA.107.653576 18347220

[20] Surkova E Cosyns B Gerber B Gimelli A La Gerche A Ajmone Marsan N. The dysfunctional right ventricle: the importance of multi-modality imaging. Eur Heart J Cardiovasc Imaging. (2022) 23(7):885–97. 10.1093/ehjci/jeac037 35234853 PMC9212350

[21] Kjaergaard J Akkan D Iversen KK Køber L Torp-Pedersen C Hassager C. Right ventricular dysfunction as an independent predictor of short- and long-term mortality in patients with heart failure. Eur J Heart Fail. (2007) 9(6-7):610–6. 10.1016/j.ejheart.2007.03.001 17462946

[22] Muraru D Onofrei A Gaia DF Mihaila S Peluso D Cucchini U Sex- and method-specific reference values for right ventricular strain by two-dimensional speckle-tracking echocardiography. J Am Soc Echocardiogr. (2018) 31(2):157–68. 10.1016/j.echo.2017.08.025

[23] Agricola E Badano L Mele D Galderisi M Slavich M Sciomer S Real-time three dimensional transesophageal echocardiography: technical aspects and clinical applications. Heart Int. (2010) 5(1):e6. 10.4081/hi.2010.e6 21977291 PMC3184702

[24] Knight DS Grasso AE Quail MA Muthurangu V Taylor AM Toumpanakis C Accuracy and reproducibility of right ventricular quantification in patients with pressure and volume overload using single-beat three-dimensional echocardiography. J Am Soc Echocardiogr. (2015) 28(3):363–74. 10.1016/j.echo.2014.10.012 25499839 PMC4346278

[25] Badano LP Kolias TJ Muraru D Abraham TP Aurigemma G Edvardsen T Standardization of left atrial, right ventricular, and right atrial deformation imaging using two-dimensional speckle tracking echocardiography: a consensus document of the EACVI/ASE/industry task force to standardize deformation imaging. Eur Heart J Cardiovasc Imaging. (2018) 19(6):591–600. 10.1093/ehjci/jey042. Erratum in: *Eur Heart J Cardiovasc Imaging*. (2018) 19(7):830–3. doi: 10.1093/ehjci/jey071. 29596561

[26] Srinivasan C Sachdeva R Morrow WR Greenberg SB Vyas HV. Limitations of standard echocardiographic methods for quantification of right ventricular size and function in children and young adults. J Ultrasound Med. (2011) 30:487–93. 10.7863/jum.2011.30.4.487 21460148

[27] López-Candales A. Right ventricular dysfunction: an independent predictor of adverse outcome in patients with myocardial infarction. Clin Cardiol. (2008) 31(5):201–2. 10.1002/clc.2020917729299

[28] Sugeng L Mor-Avi V Weinert L Niel J Ebner C Steringer-Mascherbauer R Multimodality comparison of quantitative volumetric analysis of the right ventricle. JACC Cardiovasc Imaging. (2010) 3(1):10–8. 10.1016/j.jcmg.2009.09.017 20129525

[29] Park JB Lee SP Lee JH Yoon YE Park EA Kim HK Quantification of right ventricular volume and function using single-beat three-dimensional echocardiography: a validation study with cardiac magnetic resonance. J Am Soc Echocardiogr. (2016) 29(5):392–401. 10.1016/j.echo.2016.01.010 26969137

[30] De Potter T Weytjens C Motoc A Luchian ML Scheirlynck E Roosens B Feasibility, reproducibility and validation of right ventricular volume and function assessment using three-dimensional echocardiography. Diagnostics. (2021) 11(4):699. 10.3390/diagnostics11040699 33919794 PMC8070805

[31] Papanastasiou CA Bazmpani MA Kokkinidis DG Zegkos T Efthimiadis G Tsapas A The prognostic value of right ventricular ejection fraction by cardiovascular magnetic resonance in heart failure: a systematic review and meta-analysis. Int J Cardiol. (2022) 368:94–103. 10.1016/j.ijcard.2022.08.008 35961612

[32] Couto M Souto M Martínez A Maceira A Vieira C Pumar JM Accuracy of right ventricular volume and function assessed with cardiovascular magnetic resonance: comparison with echocardiographic parameters. Clin Imaging. (2020) 59(1):61–7. 10.1016/j.clinimag.2019.10.002 31760279

[33] Soliman-Aboumarie H Joshi SS Cameli M Michalski B Manka R Haugaa K EACVI Survey on the multi-modality imaging assessment of the right heart. Eur Heart J Cardiovasc Imaging. (2022) 23(11):1417–22. 10.1093/ehjci/jeac183 36093580

